# Ablation of PI3K-p110alpha Impairs Maternal Metabolic Adaptations to Pregnancy

**DOI:** 10.3389/fcell.2022.928210

**Published:** 2022-07-01

**Authors:** Jorge Lopez-Tello, Esteban Salazar-Petres, Liam Webb, Abigail L. Fowden, Amanda N. Sferruzzi-Perri

**Affiliations:** Department of Physiology, Development and Neuroscience, Centre for Trophoblast Research, University of Cambridge, Cambridge, United Kingdom

**Keywords:** pregnancy, metabolism, mitochondria, PI3K, signalling

## Abstract

Pregnancy requires adaptations in maternal metabolism to support fetal growth. The phosphoinositol-3-kinase (PI3K) signalling pathway controls multiple biological processes and defects in this pathway are linked to metabolic disorders including insulin resistance and glucose intolerance in non-pregnant animals. However, relatively little is known about the contribution of PI3K signalling to the maternal metabolic adaptations during pregnancy. Using mice with partial inactivation of the PI3K isoform, p110α (due to a heterozygous dominant negative mutation; *Pik3ca*-D933A), the effects of impaired PI3K-p110α signalling on glucose and insulin handling were examined in the pregnant and non-pregnant states and related to the morphological, molecular, and mitochondrial changes in key metabolic organs. The results show that non-pregnant mice lacking PI3K-p110α are glucose intolerant but exhibit compensatory increases in pancreatic glucose-stimulated insulin release and adipose tissue mitochondrial respiratory capacity and fatty acid oxidation. However, in pregnancy, mutant mice failed to show the normal increment in glucose intolerance and pancreatic β-cell mass observed in wild-type pregnant dams and exhibited further enhanced adipose tissue mitochondrial respiratory capacity. These maladaptations in pregnant mutant mice were associated with fetal growth restriction. Hence, PI3K-p110α is a key regulator of metabolic adaptations that support fetal growth during normal pregnancy.

## Introduction

Successful pregnancy requires adaptive changes in maternal metabolism that increase glucose and fatty acid availability to support fetal growth (Napso et al., 2018; Sferruzzi-Perri et al., 2020). These adaptations are particularly important during late gestation when the fetus is growing most rapidly in absolute terms. More specifically, the mother becomes glucose intolerant and insulin resistant in accompaniment with decreases in the sensitivity of skeletal muscle and adipose tissue to the actions of insulin during pregnancy (Musial et al., 2016; Napso et al., 2018; Sferruzzi-Perri et al., 2020). To counteract this new state of insulin resistance, pancreatic β-cell mass expands in the mother during pregnancy (Salazar-Petres and Sferruzzi-Perri, 2022). Failure of the mother to adapt her metabolism effectively during pregnancy can cause abnormal birth-weight and metabolic diseases such as gestational diabetes mellitus (GDM) (Sferruzzi-Perri et al., 2020), with important short- and long-term consequences for the health of both the mother and offspring. Despite this, little is known about the signalling pathways governing maternal metabolic adaptations during pregnancy.

The phosphoinositol-3-kinase (PI3K) signalling pathway is one of the most important intracellular pathways regulating cell metabolism and growth (Engelman et al., 2006). It is critical for mediating the metabolic effects of insulin (Foukas et al., 2006; Knight et al., 2006; Taniguchi et al., 2006) by initiating signalling cascades that promote glucose uptake, glycogen synthesis, and cell growth, and survival (Sopasakis et al., 2010; Pérez-García et al., 2014). Class IA PI3Ks signal downstream of tyrosine kinase receptors, like the insulin receptor, and are heterodimers composed of a regulatory subunit (p85) and a catalytic subunit (p110). The catalytic isoform p110α, encoded by the gene *Pik3ca*, is ubiquitously expressed in mammals, and is essential for normal development. Its global inactivation in mice through a homozygous knock-in dominant negative mutation, *Pik3ca* ^D933A/D933A^ causes embryonic lethality (Foukas et al., 2006), whilst a single copy of the mutation in heterozygous p110α^D933A/+^ mice does not affect viability, but causes metabolic dysfunction postnatally (Foukas et al., 2006; Sferruzzi-Perri et al., 2016). Loss of PI3K-p110α specifically in adipose tissue results in mice with glucose intolerance and insulin resistance (Nelson et al., 2014; Araiz et al., 2019). In contrast, selective deletion of PI3K-p110α in the skeletal muscle has no effect on whole-body glucose tolerance or insulin sensitivity, but alters skeletal muscle mass in association with enhanced protein degradation and altered mitochondrial expression of genes involved in oxidative phosphorylation (OXPHOS) (Li et al., 2019).

While these studies indicate the importance of PI3K-p110α in controlling metabolism in the non-pregnant state (Foukas et al., 2006; Foukas et al., 2013), comparatively little is known about the role of PI3K-p110α in adaptations of maternal metabolism in response to pregnancy. Previous studies in pregnant mice have shown that alterations in PI3K-p110α abundance in skeletal muscle and adipose tissue induced by maternal obesity are associated with aberrant maternal metabolic responses and impaired fetal growth (Musial et al., 2017; Musial et al., 2019). In the current study we used p110α^D933A/+^ female mice (referred to as α/+ mutants) to investigate the significance of PI3K-p110α signalling in glucose and insulin handling, and morphological, molecular and metabolic changes in key metabolic organs (pancreas, adipose tissue and skeletal muscle) in non-pregnant and pregnant states. Overall, our study demonstrates that PI3K-p110α signalling is crucial for driving the metabolic changes that occur in the mother during pregnancy. We demonstrate that pregnant α/+ dams do not achieve the same degree of glucose intolerance nor pancreatic β-cell mass expansion as seen in wild type (WT) mice during pregnancy. These metabolic maladaptations in pregnant α/+ mice were associated with reduced fetal growth and defects in the morphological, molecular and mitochondrial phenotype of maternal skeletal muscle and adipose tissue.

## Research Design and Methods

### Ethics Statement and Mouse Work

Mice were housed in the University of Cambridge Animal Facility and all procedures were performed in accordance with the United Kingdom Home Office Animals (Scientific Procedures Act 1986). The generation and use of α/+ mice was previously described (Foukas et al., 2006; Sferruzzi-Perri et al., 2016) and experimental design is shown in Supplementary Figure S1. All animals were maintained on a C57BL/6J background (back-crossed for at least 10 generations) and identification of α/+ genotype was conducted using conventional PCR with the primers; 5′-TTC​AAG​CAC​TGT​TTC​AGC​T-3′ and 5′-TTA​TGT​TCT​TGC​TCA​AGT​CCT​A-3′ [further details in (Sferruzzi-Perri et al., 2016)]. Mice were housed in groups of 4–5 dams per cage at a room temperature of 21°C under a 12-h dark/12-h light cycle conditions and had free access to water and food (RM3 diet; Special Diet Services). At 4 months of age, virgin α/+ and WT mice were mated with WT males. The day a copulatory plug was found was denoted as day 1 of pregnancy and terminal procedures were conducted on day 18. A separate cohort of WT and α/+ mice were left un-mated and used for non-pregnant studies. A total of 26 WT (11 non-pregnant and 15 pregnant) and 20 mutant (9 non-pregnant and 11 pregnant) mice were used. Briefly, a glucose or insulin tolerance test (GTT and ITT, respectively) was performed on non-pregnant and pregnant mice (on day 17 of pregnancy). The day after the GTT/ITT, mice were killed by cervical dislocation. Adipose tissue depots, pancreas, and skeletal muscle were removed, weighed and collected for molecular analysis.

### Glucose and Insulin Tolerance Test

Non-pregnant and pregnant mice (on day 17 of pregnancy) were starved for a total of 4 h (from 10am to 2pm). Animals were intraperitoneally injected with a bolus of glucose (10% weight for volume, 1 g/kg body weight) or insulin (0.25 units/kg, human insulin, Actrapid; Novo Nordisk). Glucose concentrations were determined at 0, 15, 30, 60, and 120 min post injection with a blood glucose meter (Onetouch, Verio). For assessment of glucose-stimulated insulin secretion (GSIS), blood was collected 15 min after glucose administration in heparinised capillary tubes, centrifuged and storage at −20°C for insulin determination following manufacturer instructions (10-1247-01, Mercodia). After the metabolic test, mice were placed in their home cage with soaked diet to resume *ad libitum* feeding.

Glucose concentrations during the GTT and ITT were normalised to basal (time zero) levels, and the area under the curve calculated using the trapezoid rule. Homeostasis model assessment of insulin resistance (HOMA-IR) and steady-state β-cell function (HOMA-B), indexes were assessed using the online-based calculator on the Diabetes Trials Unit of the University of Oxford website (https://www.dtu.ox.ac.uk/homacalculator/).

### Adipose and Skeletal Muscle Mitochondrial Respirometry

Fresh adipose tissue (retroperitoneal fat) and skeletal muscle samples (*biceps femoris*) were cleaned in ice-cold x1 phosphate buffered saline and placed into ice-cold biopsy preservation medium (Sowton et al., 2020). Samples were permeabilized in respiratory medium BIOPS (Salazar-Petres et al., 2022) containing saponin (5 mg in 1 ml, Sigma-Aldrich, United Kingdom) for 20 min on ice. To remove endogenous substrates and contaminants, samples were washed three times 5 min in respiratory medium MiR05 (Salazar-Petres et al., 2022). Oxygen concentration (µM) and flux per tissue mass (pmol O_2_/s/mg) were recorded in real-time using calibrated oxygen sensors and Datlab software (Oroboros Instruments, Austria). Respiratory rates were corrected for instrumental background considering oxygen consumption of the oxygen sensor and oxygen diffusion out of, or into, the oxygraph chamber measured under experimental conditions in MiR05 medium without any tissue present.

High resolution respirometry (HRR; Oxygraph 2k respirometer; Oroboros Instruments) was used to assess the capacity for respiratory substrate use and electron transport system (ETS) function in fresh adipose and skeletal muscle, as described (Napso et al., 2022). A list of mitochondrial substrates, uncouplers and inhibitors used for the study are shown in Supplementary Table S1.

### Histological Analysis

Tissues were embedded in paraffin blocks and cut at 5 μm. Sections of pancreas were stained for insulin with a rabbit polyclonal antibody (Cell Signalling; 4590S; 1:100) to allow us to assess β-cell mass. Secondary antibody (Abcam, ab6720; 1:1000) and streptavidin-horseradish peroxidase (Rockland, S000-03, 1:500) were applied before visualising with 3,3′-diaminobenzidine (DAB; Abcam, ab64238) and nuclear fast red (Vector Laboratories, H-3403) as a counterstaining reagent. Sections were digitalized using a NanoZoomer 2.0-RS. Two types of analyses were conducted on these sections. First, in at least three non-consecutive pancreatic sections (which were separated by 100 μm), β-cell mass was measured by dividing the area of positive insulin staining (DAB staining) by the total tissue area (nuclear fast red staining) and multiplied that by the weight of the pancreas. Secondly, the size of insulin-positive islets within an area was analysed using freehand tool in ImageJ and then the number of islets that fell into the small (<1000 μm^2^), medium (1000–3000 μm^2^) and large (>3000 μm^2^) categories was determined.

Adipose tissue and skeletal muscle sections were stained with hematoxylin and eosin. Sections were digitalized using a NanoZoomer 2.0-RS. Adipocyte size was determined with Adiposoft software and pancreas islet and muscle fibre sizes were analysed with the NDP.view2 software. All analyses were conducted blind to the experimental groups.

### Western Blotting

Proteins were extracted from adipose and skeletal muscle samples using RIPA buffer. Membranes were incubated with antibodies described in Supplementary Table S2. Reactive bands were detected using an iBright Imaging systems (Thermo-Fisher) by chemiluminescence (SuperSignal West Femto, Thermo-Scientific). Signal intensity of protein bands were quantified with ImageJ software and Ponceau staining used for normalization of protein abundance (Romero-Calvo et al., 2010).

### Statistical Analysis

Statistical analysis was evaluated by unpaired Student’s *t*-test or two-way ANOVA followed by Tukey post hoc test with values of *p* < 0.05 considered significant. Feto-placental weights were analysed using litter means followed by one-way ANOVA coupled to Tukey post hoc test. GTT and ITT were analysed by repeated measured two-way ANOVA. To understand the importance of PI3K-p110α in mediating adaptations in pregnancy, we analysed the effects of PI3K-p110α deficiency (referred to as P_gen_), as well as the effects of the pregnancy on maternal physiology (referred to as P_state_). The effects of both genotype and physiological status interaction are referred throughout as P_gen*state_. All data are reported as mean ± SEM.

## Results

### PI3K-p110α Dams are Smaller in Size and Have Growth Restricted Fetuses

**FIGURE 1:**
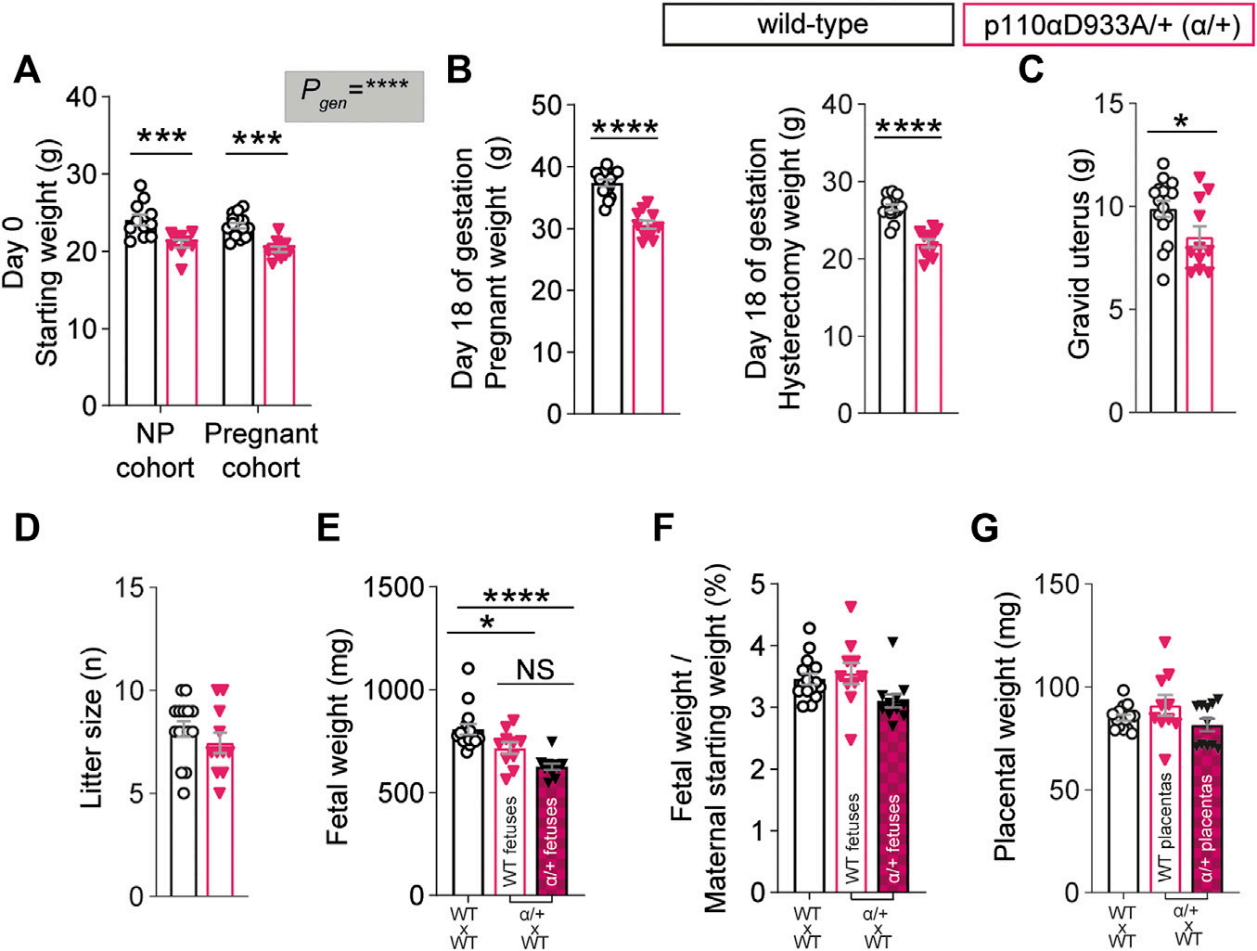
PI3K-p110α regulates maternal bodyweight and fetal growth. **(A)** Female starting weights (*n* = 9–15 mice; two-way ANOVA) **(B)** Hysterectomised weights (after removal of the uterus) of the pregnant mice (*n* = 11–15 mice; two-way ANOVA). **(C)** Weight of the gravid uterus (*n* = 11–15 mice; unpaired Student’s *t*-test). **(D)** Litter size (*n* = 11–15 mice; unpaired Student’s *t*-test). **(E)** Fetal weights obtained from 11 to 15 dams (each dot represents a litter mean; one-way ANOVA). **(F)** Fetal weight divided by maternal weight at the start of pregnancy (each dot represents a litter mean; one-way ANOVA). **(G)** Placental weight obtained from 11 to 15 dams (each dot represents a litter mean; one-way ANOVA). Data are individual-litter values and mean ± SEM.*p < 0.05; **p < 0.01; ***p < 0.001; ****p < 0.0001. NS (not significant), NP (non-pregnant).

In line with previous work (Foukas et al., 2006; Sferruzzi-Perri et al., 2016), non-pregnant α/+ mice were 13% lighter than WT (Figure 1A). After removal of the gravid uterus (hysterectomised weight), pregnant α/+ dams were 17% lighter than WT dams (Figure 1B). The weight of the gravid uterus of α/+ dams was significantly reduced by 14% compared to WT, even though litter size was unaltered (Figures 1C,D). WT fetuses from α/+ mothers were similar in weight to α/+ siblings, but both WT and α/+ fetuses in α/+ mothers were significantly lighter than WT fetuses from WT dams (Figure 1E). Moreover, analysis of fetal weights compared to maternal size (Figure 1F), indicated that the reduction in WT and α/+ fetal weights was proportional to the decreased starting weight of pregnant α/+ compared to WT dams (Figure 1A). Finally, placental weight was unaltered (Figure 1G). Taken together, these data reinforce the critical role of PI3K-p110α signalling pathway in regulating maternal body weight and fetal development.

### PI3K-p110α Regulates *In Vivo* Glucose and Insulin Homeostasis

**FIGURE 2:**
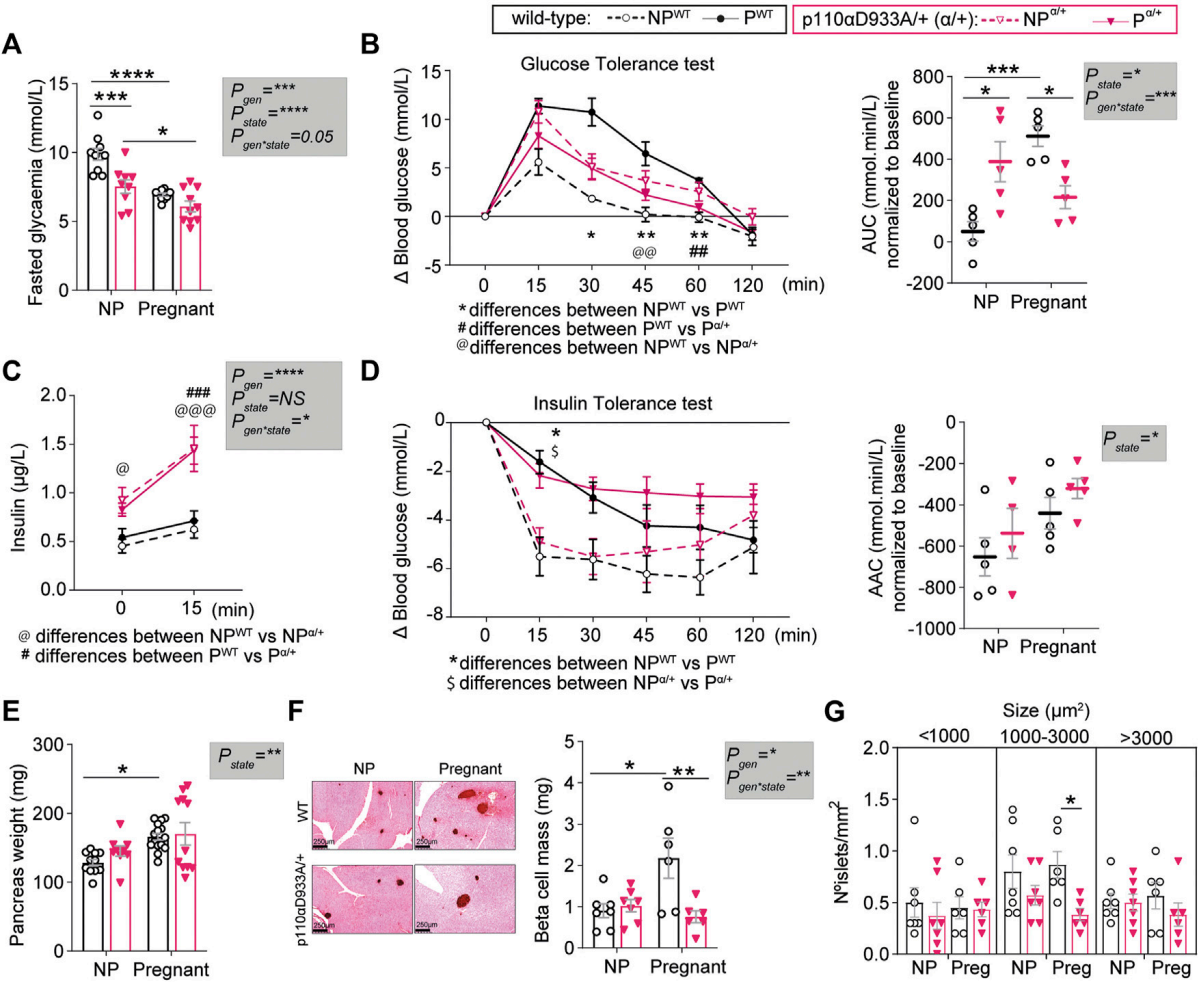
PI3K-p110α regulates glucose homeostasis and participates in the expansion of pancreatic β-cell mass in response to pregnancy. **(A)** Fasted glucose levels in non-pregnant (NP) and pregnant mice (*n* > 5/group; two-way ANOVA). **(B)** Glucose tolerance test and area under the curve (AUC) after intraperitoneal injection of glucose (*n* = 5/group; GTT curve analysed by two-way repeated measures ANOVA; AUC analysed by two-way ANOVA). **(C)** Fasted insulin levels for GTT animals before administration of glucose at time 0 and 15 min after intraperitoneal injection of glucose (*n* = 4–5/group; two-way MIXED ANOVA). **(D)** Insulin tolerance test and area above the curve (AAC) after intraperitoneal injection of insulin (*n* = 4–5/group; ITT curve analysed by two-way repeated measures ANOVA; AAC analysed by two-way ANOVA). **(E)** Pancreas weight (*n* = 9–15/group; two-way ANOVA). **(F)** Representative images of insulin staining using DAB for pancreas islet detection (scale bar = 250 μm) and β-cell mass (*n* = 6–7/group; two-way ANOVA). **(G)** Distribution of islets sizes per area analysed (*n* = 6–7/group; two-way ANOVA). Data are individual values and/or mean ± SEM. *p < 0.05; **p < 0.01; ***p < 0.001; ****p < 0.0001. For B and D, the definitions for *, @, $ and # are provided within the figure.

We then assessed glucose and insulin handling *in vivo*, as it is known that p110α deletion and pregnancy modify glucose homeostasis. Following a 4-h fast, non-pregnant α/+ mice had lower circulating glucose concentrations compared to non-pregnant WT mice (Figure 2A). In both WT and α/+ dams, glucose concentrations were lower in the pregnant than non-pregnant state (Figure 2A).

Non-pregnant α/+ mice were glucose intolerant compared to WT mice, consistent with earlier work (Foukas et al., 2006). In contrast to the pregnancy induced increase in glucose intolerance in the WT mice, there was no further increase in glucose intolerance in the α/+ mice during pregnancy (Figure 2B and Supplementary Figure S2). Consequently, α/+ mice were less glucose intolerant than WT mice during pregnancy (Figure 2B). The α/+ mice were hyperinsulinemic compared to non-pregnant WT mice, in both the non-pregnant and pregnant states (time 0, Figure 2C). Insulin secretion in the glucose-stimulated state [measured 15 min post-administration of glucose; glucose-stimulated insulin secretion (GSIS)] was greater in both non-pregnant and pregnant α/+ mice compared to WT (Figure 2C). We did not observe a significant effect of α/+ genotype on insulin sensitivity in either state; both WT and α/+ mice became equally insulin insensitive during pregnancy (Figure 2D). Finally, insulin resistance index (HOMA-IR) and pancreatic *β* cell function index (HOMA-*β*), were overall elevated in α/+ mice, with pairwise comparisons revealing a significant effect in the non-pregnant state (Supplementary Figure S2). These data indicate that PI3K-p110α controls glucose and insulin dynamics both in non-pregnant and pregnant states. Moreover, intact PI3K-p110α signalling is required to fully achieve the normal level of maternal glucose intolerance during pregnancy.

### PI3K-p110α is Required for Pancreatic β-Cell Mass Expansion

To understand changes in the relationship between glucose and insulin in non-pregnant and pregnant state with the α/+ genotype, we analysed the morphology of the pancreas. Pancreas weight and β-cell mass did not vary between non-pregnant α/+ and WT mice (Figures 2E,F). During pregnancy, pancreas weight and β-cell mass increased in WT mice by 23% and 60%, respectively (Figure 2E). These changes were not observed in the pregnant α/+ mice with the consequence that β-cell mass was significantly lower pregnant α/+ than WTs mice (−65%, Figure 2F). Closer analysis of islet size distribution revealed that pregnant α/+ mice had fewer medium sized islets (1000–3000 µm^2^) compared to pregnant WTs (Figure 2G). Collectively, these data demonstrate that PI3K-p110α signalling is required for pancreatic β-cell mass expansion in the mother during pregnancy.

### Deficiency in PI3K-p110α Impairs Adipose Tissue Expansion With Changes in Insulin Signalling and Metabolic Proteins

**FIGURE 3:**
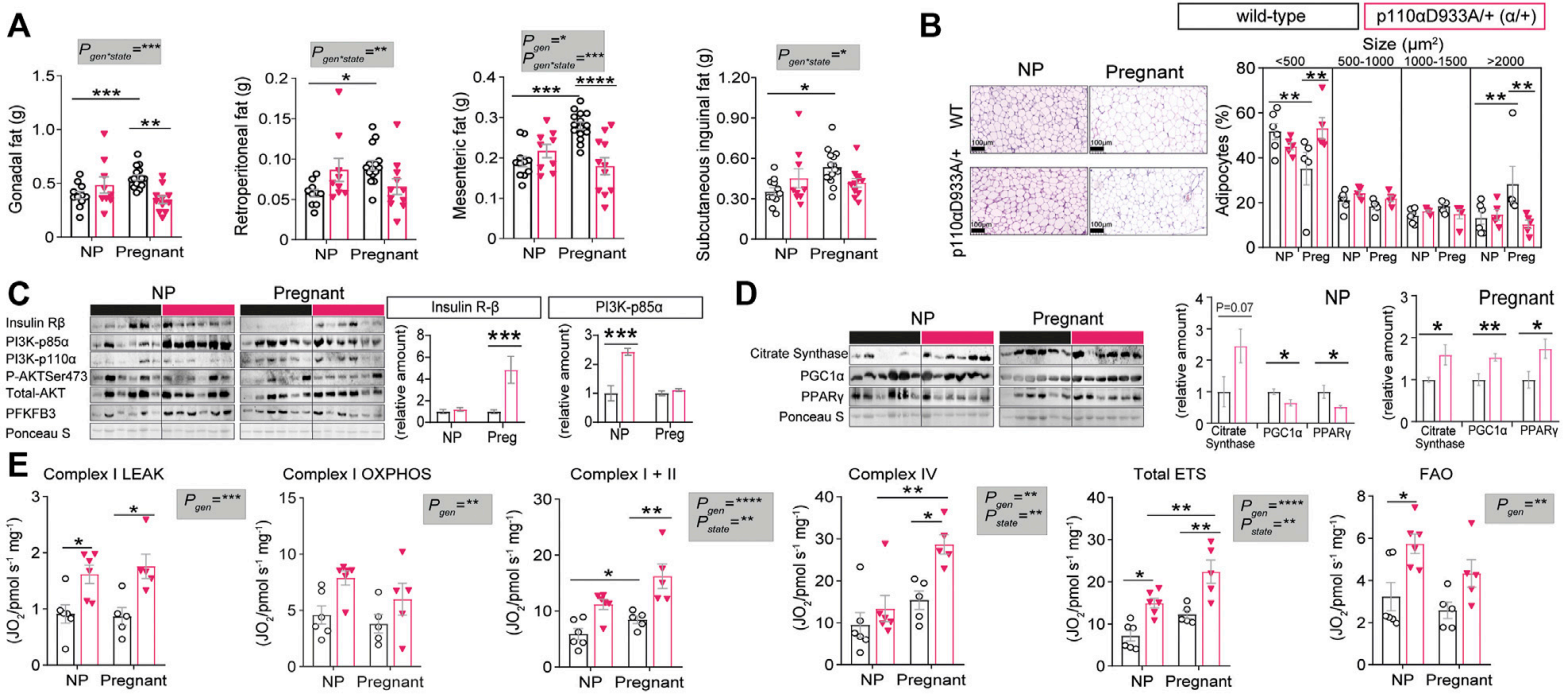
PI3K-p110α regulates adiposity levels and mitochondrial function. **(A)** Adipose depot weights in non-pregnant (NP) and pregnant mice (*n* = 9–15/group; two-way ANOVA). **(B)** Histological analysis of adipocyte size (*n* = 5–6/group; two-way ANOVA) (scale bar = 100 μm). **(C)** Western blot analysis of metabolic signalling proteins: insulin receptor β subunit (Insulin Rβ), phosphoinositide-3-kinase subunits (PI3K-p85α and PI3K-p110α), total and phosphorylated protein kinase B (Total AKT and P-AKTSer473) and 6-phosphofructo-2-kinase/fructose-2,6-biphosphatase 3 (PFKFB3). (*n* = 6/group; un-paired Student’s *t*-test). **(D)** Western blot analysis of mitochondrial related proteins; citrate synthase, peroxisome proliferator-activated receptor gamma coactivator 1-alpha (PGC1α) and peroxisome proliferator-activated receptor gamma (PPAR-γ). (*n* = 6/group; un-paired Student’s *t*-test). **(E)** Mitochondrial respiration rates. Information about substrate/inhibitor in relation to complex activation can be found in Supplementary Table S1. Maximum electron transfer system capacity (Total ETS) and fatty acid oxidation (FAO). (*n* = 5–6/group; two-way ANOVA). Data in **(B–E)** are from the retroperitoneal fat pad. Data are individual values and/or mean ± SEM. *p < 0.05; **p < 0.01; ***p < 0.001; ****p < 0.0001.

We analysed specific fat pads to gain further insights into the mechanisms underlying pregnancy adaptations and the metabolic impact of α/+ genotype. No significant differences in fat pad sizes were observed between non-pregnant α/+ and WT mice (Figure 3A). In WT mice, pregnancy induced expansion of all the fat pads studied (+30% gonadal; +37.5% retroperitoneal; +32% mesenteric; +34% subcutaneous inguinal fat). However, this pregnancy-induced expansion failed to occur in α/+ dams. Moreover, the weights of gonadal and mesenteric fat depots were significantly lower in α/+ than WT pregnant dams (−37% and −32%, respectively) (Figure 3A). Further analysis of the retroperitoneal fat showed no differences in adipocyte size between non-pregnant α/+ and WT mice (Figure 3B). However, WT females showed a reduced percentage of small adipocytes (<500 µm^2^), but increased percentage of large adipocytes (>2000 µm^2^) in response to pregnancy. This pregnancy-related adipocyte expansion, however, was not observed in α/+ mice. In addition, compared to pregnant WTs, pregnant α/+ dams had a more <500 µm^2^ and less >2000 µm^2^ adipocytes (Figure 3B). These data indicate that PI3K-p110α signalling is required for adipose expansion during pregnancy.

To explore the molecular mechanisms related to the changes in adipose tissue and glucose tolerance in α/+ mice during pregnancy, abundance of key insulin-PI3K signalling proteins were quantified in the retroperitoneal fat of non-pregnant and pregnant WT and α/+ mice. Whilst adipose tissue insulin receptor-β abundance was similar in non-pregnant WT and α/+ animals, pregnant α/+ showed increased abundance of the receptor when compared to pregnant WT mice (Figure 3C). Protein levels of the PI3K regulatory subunit p85α were significantly elevated in α/+ versus WT mice in the non-pregnant state, but not in pregnancy (Figure 3C). No significant differences were detected in the abundance of PI3K-p110α and total protein levels of AKT, including the level of activated phosphorylated AKT at Ser473, or in the abundance of PFKFB3, a master regulator of adipocyte nutrient metabolism (Huo et al., 2010) (Figure 3C). These results indicate that loss PI3K-p110α activity triggers compensatory changes in other PI3K isoforms and insulin receptor levels. Moreover, increased adipose tissue insulin sensitivity (*via* increased insulin receptor) may explain, in part, the altered glucose tolerance of α/+ relative to WT dams in late pregnancy.

### PI3K-p110α is a Key Regulator of Adipose Mitochondrial Related Protein Abundance and Respiratory Capacity

Since recent reports show that changes in PI3K-p110α signalling can affect mitochondrial efficiency and function (Nelson et al., 2014; Li et al., 2019), we measured proteins related to mitochondrial function. The retroperitoneal fat of non-pregnant α/+ mice had a similar citrate synthase abundance (indicator of mitochondrial density), but ≈40% lower PGC1α (mitochondrial biogenesis) and PPARγ (mitochondrial biogenesis and lipid handling) abundance compared to non-pregnant WT counterparts (Figure 3D). However, in pregnancy, all three proteins were significantly elevated by >40% in the adipose tissue of α/+ compared to WT dams (Figure 3D). We then analysed mitochondrial bioenergetics using HRR. This showed that, compared to WTs, α/+ mice had elevated oxygen consumption rates with all the substrate combinations irrespective of whether they were pregnant or not (Figure 3E). Pregnancy increased oxygen consumption through Complex I, Complex IV and Total ETS due predominantly to effects in the α/+ mice (Figure 3E). Finally, oxygen consumption associated with fatty acid oxidation (FAO) was significantly elevated in α/+ females compared to WTs, but this increment was due predominantly to an effect in the non-pregnant state (Figure 3E). Collectively, these data indicate that a deficiency in PI3K-p110α signalling results in altered mitochondrial function and respiration in adipose tissue both in non-pregnant and pregnant states.

### Deficiency in PI3K-p110α Alters Skeletal Muscle Size, Insulin Signalling and Glycolytic Ability

**FIGURE 4:**
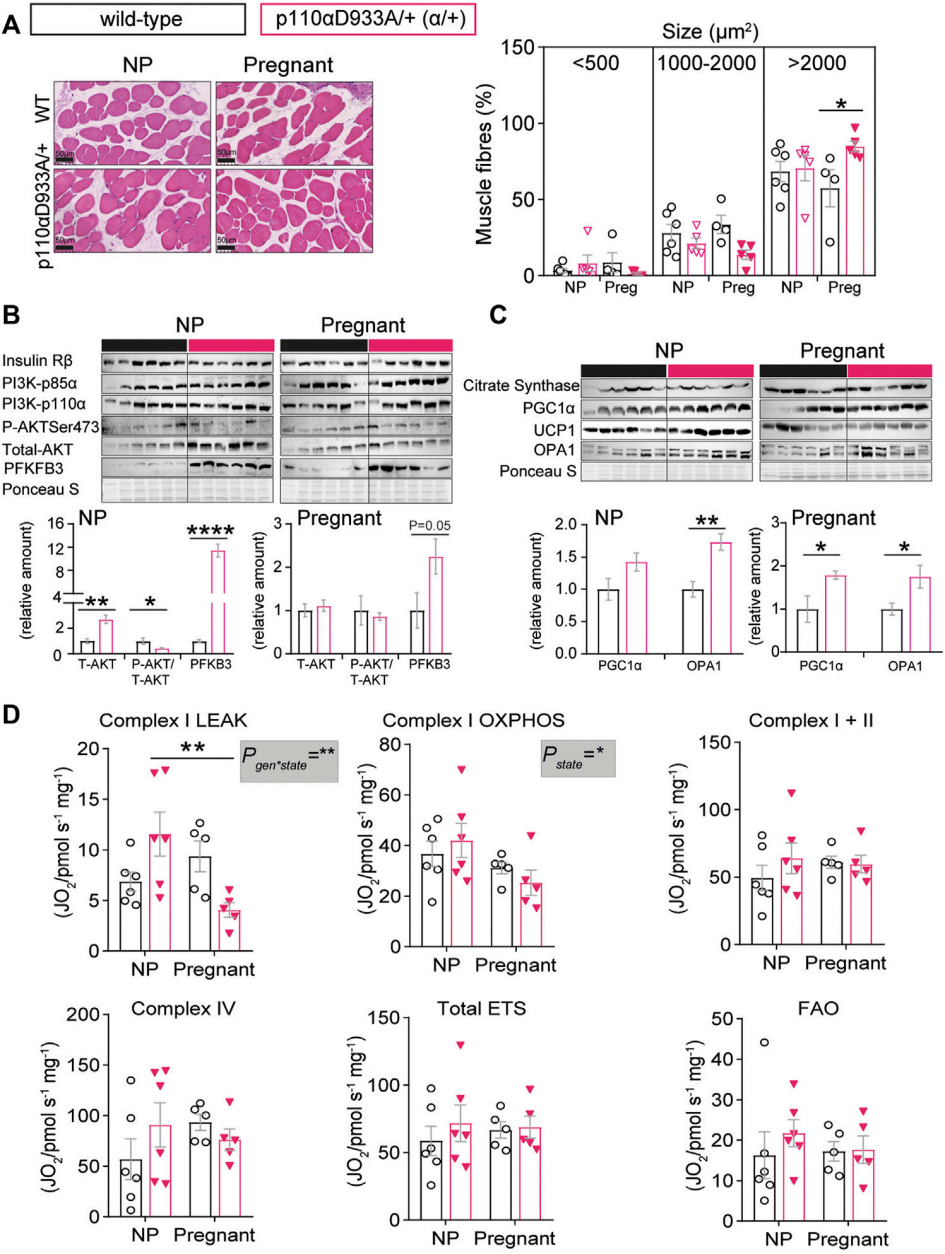
PI3K-p110α regulates myofibre size and mitochondrial metabolism. **(A)** Skeletal muscle fibre size in non-pregnant (NP) and pregnant mice (*n* = 4–6/group; two-way ANOVA) (scale bar = 50 μm). **(B,C)** Western blot analysis of metabolic signalling pathways in the skeletal muscle: insulin receptor β subunit (Insulin Rβ), phosphoinositide-3-kinase subunits (PI3K-p85α and PI3K-p110α), total and phosphorylated protein kinase B (Total AKT and P-AKTSer473) and 6-phosphofructo-2-kinase/fructose-2,6-biphosphatase 3 (PFKFB3), peroxisome proliferator-activated receptor gamma coactivator 1-alpha (PGC1α), mitochondrial brown fat uncoupling protein 1 (UCP1) and mitochondrial dynamin like GTPase (OPA1) (*n* = 6/group; un-paired Student’s *t*-test). **(D)** Mitochondrial respirometry (*n* = 5–6/group; two-way ANOVA). Information about substrate/inhibitor in relation to complex activation can be found in Supplementary Table S1. Maximum electron transfer system capacity (Total ETS) and fatty acid oxidation (FAO). Data are individual values and/or mean ± SEM. *p < 0.05; **p < 0.01; ***p < 0.001; ****p < 0.0001.

To further investigate the role of PI3K-p110α in the metabolic control during pregnancy, we assessed skeletal muscle fibre size and insulin signalling abundance. We found no significant differences in myofiber sizes between non-pregnant WT and α/+ mice (Figure 4A). However, compared to pregnant WT mice, pregnant α/+ had an increased number of large myofibers (defined as >2000 µm^2^) in their *biceps femoris* (Figure 4A). Investigation of the insulin signaling pathway revealed skeletal muscle abundance of total AKT and phosphorylated activated AKT-Ser473 was greater and less, respectively, in non-pregnant α/+ than WT mice (Figure 4B). Moreover, relative to WT, non-pregnant α/+ females had increased levels of PFKFB3, an enzyme critical in glycolysis in skeletal muscle (Winder et al., 1994) (Figure 4B). However, the abundance of AKT, and its activation status, did not differ between pregnant WT and α/+ dams although PFKFB3 showed a tendency to remain increased in the pregnant α/+ dams compared to WT (*p* = 0.05, Figure 4B). Compared to WT, no significant differences were observed in the abundance of insulin receptor-β, PI3K-p85α or PI3K-p110α in α/+ mice in either the non-pregnant or pregnant state. Thus, in the non-pregnant state, dysregulated skeletal muscle insulin signalling in the α/+ mice may be related to their glucose intolerance. Additionally, intact PI3K-p110α signalling is a key regulator of muscle fibre composition during pregnancy, but this is unrelated to any apparent change in the insulin signalling pathway.

### PI3K-p110α Affects Skeletal Muscle Mitochondrial Dynamic Proteins and Respiratory Capacity

Citrate synthase abundance was unaffected by genotype or pregnancy (Figure 4C). However, PGC1α was upregulated in pregnant α/+ dams compared to their respective WT controls. Skeletal muscle is rich in proteins like UCP1 and OPA1, important for coupling oxygen consumption with ATP production and mitochondrial fusion, respectively. Indeed, dysregulation of these two proteins in skeletal muscle leads to mitochondrial dysfunction (Couplan et al., 2002; Tezze et al., 2017). We found that UCP1 abundance was unaffected, while OPA1 was significantly elevated, in both non-pregnant and pregnant α/+ dams relative to their respective WT controls (Figure 4C). Using HRR, only complex I oxygen consumption rate in LEAK state was affected by α/+ genotype in the skeletal muscle. There was also a significant interaction between α/+ genotype and pregnancy state with a reduction in complex I LEAK during pregnancy in the α/+, but not WT mice (Figure 4D). Taken together, these data suggest that PI3K-p110α deficiency results in specific mitochondrial-related proteins changes but minimal effects on mitochondrial respirometry.

## Discussion

Using combination of *in vivo* genetic manipulation, whole body testing and molecular methods, this is the first study to demonstrate that PI3K-p110α is key for mediating appropriate metabolic adaptations in the mother during pregnancy that support normal fetal development. In particular, α/+ female mice failed to acquire the normal level of glucose intolerance and expand their pancreas β-cell mass during pregnancy. Moreover, loss of function in the PI3K-p110α signalling in α/+ mice was associated with changes in maternal adipose tissue and skeletal muscle morphology, insulin signalling, and mitochondrial respiratory capacity. Our data confirms that the PI3K-p110α signalling pathway functions in key metabolic tissues of the mother to favour fetal nutrient provision and ultimately, healthy growth. Together, these findings may have relevance for understanding the pathways leading to pregnancy complications, like gestational diabetes and abnormal birthweight, which can arise from maternal metabolic maladaptations in pregnancy*.*

Our *in vivo* analysis demonstrate that non-pregnant α/+ mice were glucose intolerant, suggesting that α/+ mice entered in pregnancy with an already established level of glucose intolerance compared to WT dams. However, α/+ dams did not become further glucose intolerant during pregnancy and compared to pregnant WT, were more glucose tolerant. Failure of the mother to achieve the correct level of glucose intolerance in pregnancy would be expected to limit glucose transfer to the fetus with negative effects on fetal growth. We have shown that our pregnant PI3K-p110α mutant mice transferred less glucose to both WT and mutant fetuses *in vivo,* and this is coupled to reduced fetal growth (Sferruzzi-Perri et al., 2016 and current study). Other work has also shown that inhibition of glucose transport to the fetus through specific genetic loss of glucose transporter expression leads to fetal growth restriction (Ganguly et al., 2007). Finally we have also found there are alterations in placental labyrinth structure that affect substrate supply capacity and fetal growth outcomes in PI3K-p110α mutant mice (López-Tello et al., 2019; Sferruzzi-Perri et al., 2016). However, analysis of fetal weights relative to maternal size revealed that the reduction in WT and α/+ fetal weights was proportional to the decreased starting weight of pregnant α/+ compared to WT dams. Therefore, the cause of fetal growth restriction in our PI3K-p110α mutant mice is likely a combination of maternal (e.g., size, metabolic adaptations) and placental (e.g., function and transport) defects. Future work is required to directly test the role of each individual factor in fetal growth outcomes in PI3K-p110α mutant pregnancies, although it should be acknowledged that this venture will be challenging.

Our study found that both non-pregnant and pregnant mutant mice showed enhanced GSIS, but similar insulin sensitivity compared to WT mice. This is surprising, as impaired glucose tolerance is often associated with a deficit in β-cell insulin secretion and peripheral insulin resistance (Goyal et al., 2021). It is known that inhibition of the PI3K-AKT signalling can enhance GSIS *via* changes in the intracellular insulin granule formation and fusion dynamics (Aoyagi et al., 2012). Partial inactivation of p110α potentiates β-adrenergic signalling (Araiz et al., 2019), and stimulation of insulin release is partially mediated through β-adrenergic receptors (Philipson, 2002). Therefore, consistent with the hyperinsulinaemia seen in the mutant mice, these observations may suggest a potential interaction between PI3K-p110α and β-adrenergic signalling in the release of insulin by pancreatic β-cells. Future experiments should explore this notion by performing insulin secretion experiments in islets isolated from α/+ mice.

PI3K-p110α is expressed by the murine pancreas (Baer et al., 2014), and plays a dominant role in the promotion of β-cell proliferation in response to a variety of hormonal cues in both non-pregnant (Jiang et al., 2018) and pregnant states (Salazar-Petres and Sferruzzi-Perri, 2022). Previous work reported that α/+ mice have greater β-cell area compared to WTs, reflecting an attempt to compensate for their insulin resistance state (Foukas et al., 2006). However, we did not observe significant changes in pancreatic β-cell mass between non-pregnant α/+ and WT mice. The discrepancy between the current and prior work likely relates to the age of the animals studied. For instance, our work was conducted on 4 months of age α/+ animals, whilst prior work by others was performed on animals at 11–12 weeks of age (Foukas et al., 2006). In addition, prior work has shown that α/+ mice do not show the age-related hyperinsulinemia and impairments in glucose homeostasis that are seen in WT animals (Foukas et al., 2006; Foukas et al., 2013)—highlighting that age of the animals is important when assessing metabolic physiology. A lack of a difference in β-cell mass in our study is also in line with the observation that our non-pregnant α/+ females did not show reduced whole body insulin sensitivity. The significance of failed β-cell mass growth for maternal or fetal outcomes in our pregnant α/+ animals is unknown. In pregnant rodents, studies have suggested that β-cell mass growth occurs to compensate for the reduced insulin sensitivity (Parsons et al., 1992; Sorenson and Brelje, 1997). This expansion is facilitated by the production of placental hormones, namely prolactin and placental lactogen, which signal *via* prolactin receptors and the STAT5, MAPK, and PI3K pathways to induce β-cell proliferation (Huang et al., 2009; Rieck and Kaestner, 2010; Salazar-Petres and Sferruzzi-Perri, 2022). Downstream of placental hormones, there are also changes in the expression of local islet regulators, like serotonin and hepatocyte growth factor, which are instrumental for maternal β-cell mass expansion and insulin secretion in pregnancy (Huang et al., 2009; Rieck and Kaestner, 2010; Salazar-Petres and Sferruzzi-Perri, 2022). Prior work has demonstrated that the expression of the prolactin and placental lactogen genes (*Prl3b1* and *Prl8a8*) by the placenta is significantly reduced in pregnant α/+ mice compared to WTs (Sferruzzi-Perri et al., 2016). Thus, it is likely that both reduced placental lactogen production and maternal PI3K signalling impairment underlies the failed pancreatic β-cell mass expansion observed in pregnant α/+ mice. Future work should evaluate interactions between placental hormone production and maternal PI3K signalling in modulating β-cell responses to glucose homeostasis changes during pregnancy. Indeed, secretome analysis of the mouse placenta has recently revealed that several factors could participate in maternal β-cell adaptations (Napso et al., 2021), including *via* crosstalk with other organs, like the maternal adipose tissue (Napso et al., 2018; Napso et al., 2021; Qiao et al., 2021).

The adipose tissue is one of the most important metabolic organs of the body, regulating energy homeostasis by acting as a caloric reservoir, controlling lipid levels, and producing metabolically-active hormones (Choe et al., 2016). Previous studies have shown that PI3K-p110α is involved in adipocyte differentiation (Zhao et al., 2006; Kim et al., 2009), and defects in PI3K-p110α signalling affect adiposity levels (Foukas et al., 2006; Foukas et al., 2013; Nelson et al., 2014; Ortega-Molina et al., 2015; Araiz et al., 2019). In this regard, it was previously reported that α/+ mice have increased adiposity as young adults, but reduced adiposity in later life (Foukas et al., 2006; Foukas et al., 2013). Moreover, adipose tissue-specific deletion of PI3K-p110α, using aP2-Cre or Adipoq–Cre lines, have shown contradictory results in terms of gain *versus* loss of adiposity (Nelson et al., 2014; Araiz et al., 2019). In our study, we did not see significant changes in the size of the different fat depots analysed between non-pregnant α/+ and WT mice. However, α/+ mice failed to expand their fat depots in response to pregnancy, with gonadal and mesenteric fat masses smaller in pregnant α/+ *versus* WT mice. In non-pregnant α/+ mice, PI3K-p85α was significantly overexpressed in the adipose, which is consistent with other work showing compensatory responses of different class IA PI3Ks in PI3K-p110α mutants (Bi et al., 1999; Fruman et al., 2000). Prior investigations have also shown that PI3K-p85α over-expression can interfere with the binding of PI3Ks to other proteins and negatively affect insulin-stimulated glucose uptake (Sharma et al., 1998; Bi et al., 1999). Hence, our finding of upregulated adipose PI3K-p85α expression may explain the glucose intolerance seen in non-pregnant α/+ mice. Whilst PI3K-p85α abundance was no longer altered in pregnant α/+ mice, expression of the insulin receptor-β was increased, in-line with the failed attainment of the normal degree of glucose intolerance seen during WT pregnancy. As previously mentioned, enhanced maternal tissue insulin sensitivity would be expected to reduce the availability of glucose in the mother for transfer to the fetus for growth, which is consistent with our prior work (Sferruzzi-Perri et al., 2016). Although the weight of the α/+ fetuses was not significantly different to WT littermates in the current study focused on maternal metabolic adaptations, prior work in larger cohorts of mice have reported these mutants to be growth restricted (Foukas et al., 2006; Sferruzzi-Perri et al., 2016; López-Tello et al., 2019).

Mitochondrial respiratory capacity was elevated in the adipose tissue of both non-pregnant and pregnant α/+ compared to WT mice. These findings are consistent with previous work demonstrating increased energy expenditure and mitochondrial activity when insulin signalling is selectively ablated in fat depots of mice (Araiz et al., 2019). However, as informed by the current study, the effect of α/+ genotype on adipose mitochondrial activity appeared to synergise with the pregnancy state, as total ETS and complex IV oxygen consumption rates were greatest in pregnant α/+ dams. Moreover, despite enhanced mitochondrial respiration in both states, the levels of PGC1α and PPARγ were downregulated and mitochondrial abundance (citrate synthase) only tended to be increased in the adipose of non-pregnant α/+ mice, yet all three markers (PGC1α and PPARγ levels and mitochondrial density) were increased in pregnant α/+ mice (Fernandez-Marcos and Auwerx, 2011; Mayeur et al., 2013). The upregulation of adipose PGC1α and PPARγ abundance in pregnant mutant animals may also result an increased lipid mobilization and consequently, could explain their reduced fat mass. Indeed, adipose tissue FAO was overall increased in α/+ mice. However, adipose related FAO was enhanced only in non-pregnant α/+ mice. Thus, further studies are required to understand how mitochondrial respiratory capacity, and molecular marker expression interact to affect adipose tissue changes in the context of reduced PI3K-p110α signalling and pregnancy.

Compared to the adipose, there were predominately divergent effects of α/+ on the skeletal muscle morphology, insulin signalling and mitochondrial respiratory capacity. Skeletal muscle fibre size was not affected by α/+ in non-pregnant mice, although there was a greater proportion of enlarged muscle fibres in pregnant α/+ mice, compared to pregnant WTs. In non-pregnant α/+ mice, there was also increased abundance of total AKT protein, but downregulation of AKT activity (phosphorylation) in the skeletal muscle. Compromised skeletal muscle PI3K signalling and reduced AKT activity have been coupled to diminished glucose uptake (Luo et al., 2006). Hence, alterations in AKT activity in non-pregnant α/+ mice may have contributed to their reduced glucose tolerance (Cho et al., 2001; Foukas et al., 2006). The skeletal muscle of α/+ non-pregnant mice also had increased protein levels of PFKFB3, which was unaltered in the adipose tissue, and would favour glycolysis and could affect whole body metabolism. In pregnancy, activation of AKT was no longer affected, but PFKFB3 tended to remain upregulated. Only minor differences on the mitochondrial respiratory capacity of non-pregnant and pregnant α/+ mice were seen. Interestingly, OPA1 was elevated in both non-pregnant and pregnant α/+ mice. Deletion of OPA1 in skeletal muscle results in mitochondrial dysfunction due to an increased endoplasmic reticulum stress (Pereira et al., 2017). Moreover, in humans with insulin resistance and obesity, the levels of OPA1 in the skeletal muscle are reduced compared to healthy patients (Houzelle et al., 2021). Abundance of PGC1α was also elevated in the skeletal muscle of pregnant α/+ mice. Other work has reported that PGC1α expression is induced in muscle-specific PI3K-p110α knockout mice (Li et al., 2019). Therefore, additional studies are required to assess the interactions between skeletal muscle glycolysis, OXPHOS capacity, and molecular mediators with whole glucose homeostasis and adiposity in response to altered PI3K-p110α signalling and pregnancy.

In summary, our study demonstrates PI3K-p110α signalling is involved in mediating metabolic changes in the mother during pregnancy (Supplementary Figure S3). Such changes are dependent on the tissue studied, with differential effects on growth, insulin production/signalling, glycolytic metabolism and/or mitochondrial respiratory function in the pancreas, adipose tissue, and skeletal muscle of α/+ mice. Our data also shows that defects in the metabolic physiology of α/+ mice (e.g., size, glucose handling, insulin sensitivity) have consequences for nutrient partitioning between maternal peripheral tissues and fetal requirements for growth. Alterations in the ability of the mother to adapt her metabolism in pregnancy can lead to gestational diabetes and abnormal fetal growth with long-term consequences for the disease susceptibility of mother and her child. Our studies may therefore have relevance for understanding the development of such conditions and how organ specific manipulation of PI3K-p110α signalling could offer some therapeutic benefit. Our studies also have relevance for understanding the control of growth and metabolic disorders, more generally*.*

## Data Availability

The original contributions presented in the study are included in the article/Supplementary Material, further inquiries can be directed to the corresponding authors.

## Supplementary Material

Supplementary Figure S1 Study design. Abbreviations: bp (base pairs), GTT (glucose tolerance test), ITT (insulin tolerance test), NP (non-pregnant), WT (wildtype). Gestational length in mice ∼ 20 days.

Supplementary Figure S2 Raw values for glucose and insulin tolerance tests (GTT, ITT) and homeostatic model assessment (HOMA) models. **(A,B)** Glucose values not normalized to baseline (time 0). (*n*=4–5/group; data analysed by two-way repeated measures ANOVA). #,*, @ defined within the figure. **(C,D)**. HOMA models for insulin resistance (HOMA-IR) and β-cell function (HOMA-β) (*n*=4–5/group; two-way ANOVA). Abbreviations NP (non-pregnant), P (pregnant).

Supplementary Figure S3 Main findings of the study.
