# Supplementary material for: Ablation of PI3K-p110alpha Impairs Maternal Metabolic Adaptations to Pregnancy
Source: Front Cell Dev Biol. 2022 Jul 1;10:928210. doi: 10.3389/fcell.2022.928210 (PMC9283861; doi:10.3389/fcell.2022.928210)
Supplement: Supplementary file 1 [file Table1.DOCX]

| Supplementary Table 1 | | | |
| --- | --- | --- | --- |
| Sequence | Substrate/inhibitor | Complex evaluation | Concentration |
| 1 | Malate | CI leak (activation) | 2 mM |
| 2 | Octanoyl carnitine | CI + FAO leak  (activation) | 0.2 mM |
| 3 | ADP | CI + FAO oxphos (activation) | 5 mM |
| 4 | Glutamate | CI + FAO oxphos  (activation) | 10 mM |
| 5 | Succinate | CI + CII + FAO oxphos (activation) | 10 mM |
| 6 | FCCP | CI + CII + FAO (uncoupler) | 3 X 0.25 µM |
| 7 | Rotenone | CI (inhibitor) | 0.5 µM |
| 8 | Malonic acid | CII (inhibitor) | 5 mM |
| 9 | Myxothiazol | CIII (inhibitor) | 0.5 µM |
| 10 | Antimycin A | CIII (inhibitor) | 2.5 µM |
| 11 | Ascorbate | CIV (activation) | 2 mM |
| 12 | TMPD | CIV (activation) | 0.5 mM |
| 13 | Azide | CIV (inhibitor) | 200 mM |
| Abbreviations. ADP: Adenosine diphosphate, CI: Complex I; CII: Complex II, CIII: Complex III, CIV: Complex IV, FAO: Fatty acid oxidation, FFCP: carbonyl cyanide p-triflouromethoxyphenyl hydrazone, TMPD: 2-Chloro-4,4,5,5-tetramethyl-1,3,2-dioxaphospholane. | | | |

**Supplementary Table 1.** List of substrates and inhibitors used for mitochondrial respirometry

| Supplementary Table 2 | | | | |
| --- | --- | --- | --- | --- |
| **Protein of interest** | **Species** | **Company** | **Catalogue #** | **Dilution** |
| Insulin Receptor B | Rabbit | Santa Cruz | sc-711 | 1:200 |
| PI3K-P85A | Rabbit | Milipore | 06-195 | 1:5000 |
| PI3K-P110A | Rabbit | Cell Signaling | 4249 | 1:1000 |
| Phospho-AKT (Ser473) | Rabbit | Cell Signaling | 9271 | 1:1000 |
| Total AKT | Rabbit | Cell Signaling | 9272 | 1:1000 |
| PFKFB3 | Rabbit | Cell Signaling | 13123 | 1:1000 |
| Citrate synthase | Rabbit | Abcam | ab96600 | 1:1000 |
| OPA1 | Rabbit | Cell Signaling | 80471 | 1:1000 |
| PGC1A | Rabbit | Santa Cruz | SC-13067 | 1:200 |
| PPARG | Mouse | Santa Cruz | SC-7273 | 1:200 |
| UCP1 | Rabbit | Santa Cruz | 72298 | 1:1000 |
| Amersham ECL Rabbit IgG, HRP-linked | | Cytiva | NA934 | 1:10000 |
| Amersham ECL Mouse IgG, HRP-linked | | Cytiva | NA931 | 1:10000 |

**Supplementary Table 2.** List of antibodies used for western blot
