# Supplementary figures and images for: Ablation of PI3K-p110alpha Impairs Maternal Metabolic Adaptations to Pregnancy
Source: Front Cell Dev Biol. 2022 Jul 1;10:928210. doi: 10.3389/fcell.2022.928210 (PMC9283861; doi:10.3389/fcell.2022.928210)

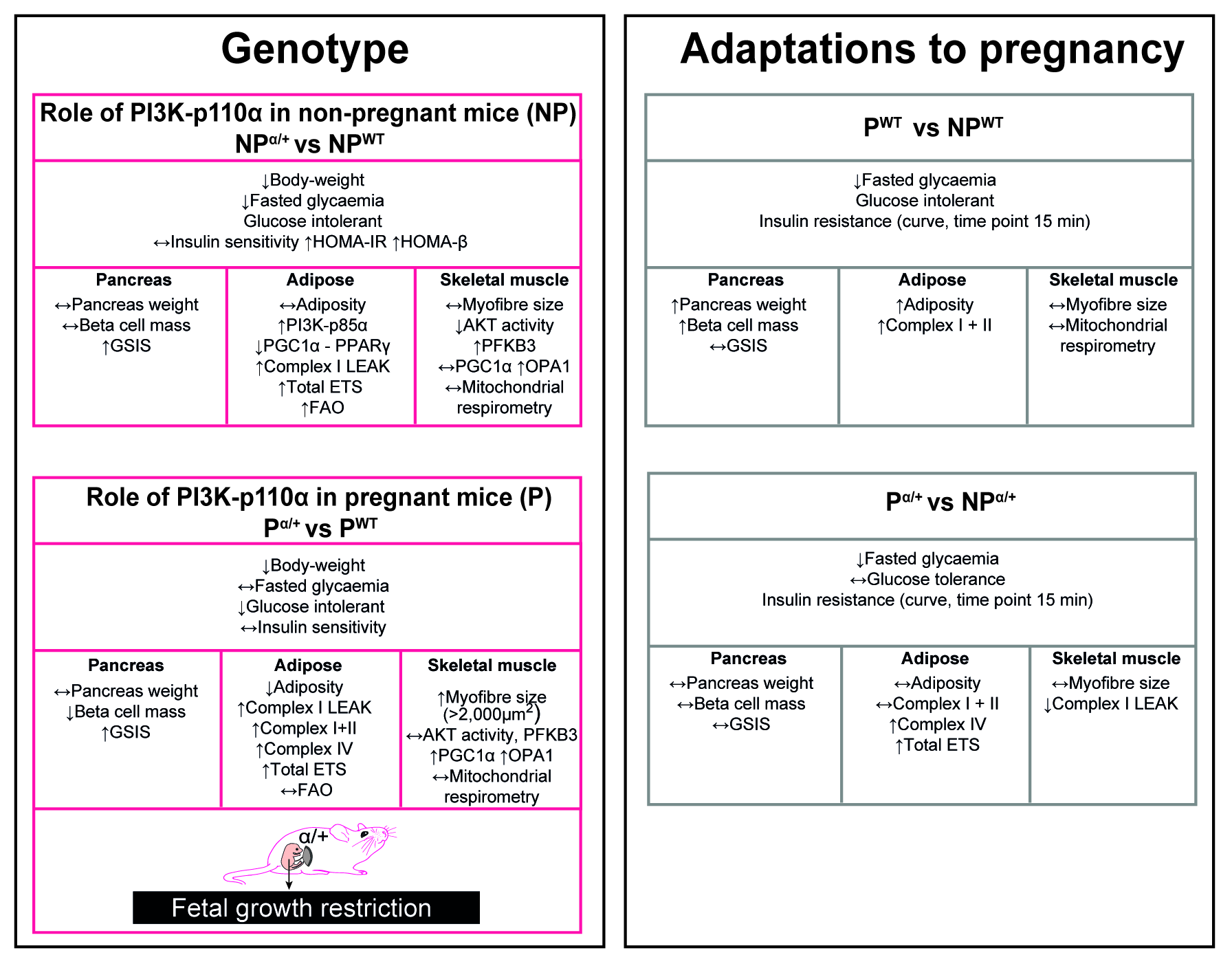

Supplement: Supplementary file 2 [file Image3.TIF]

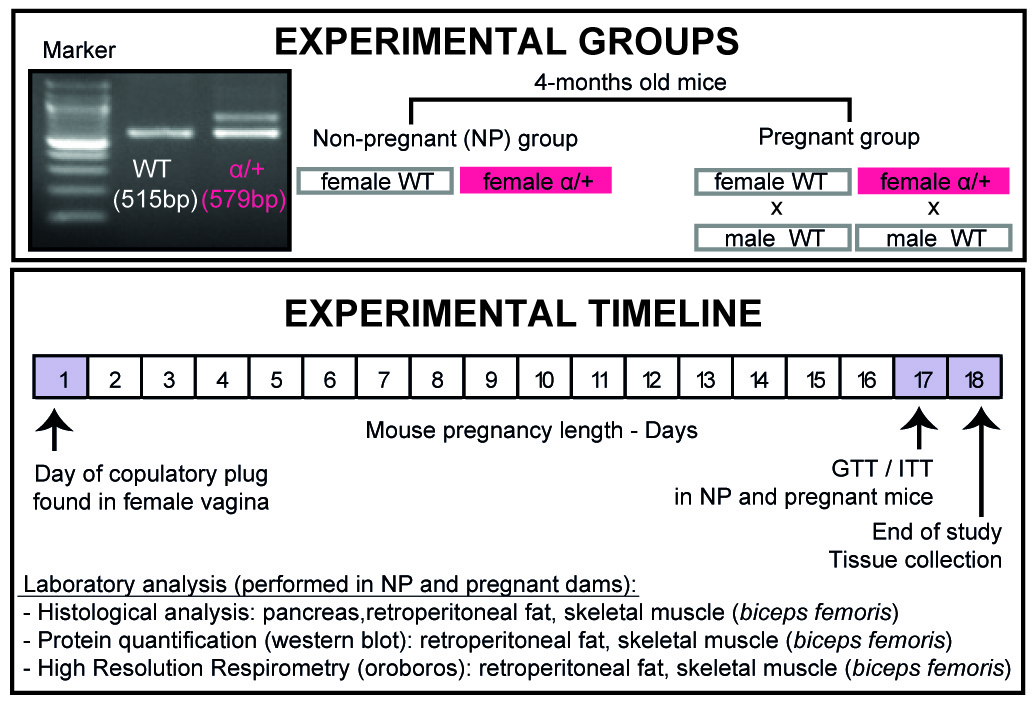

Supplement: Supplementary file 3 [file Image1.JPEG]

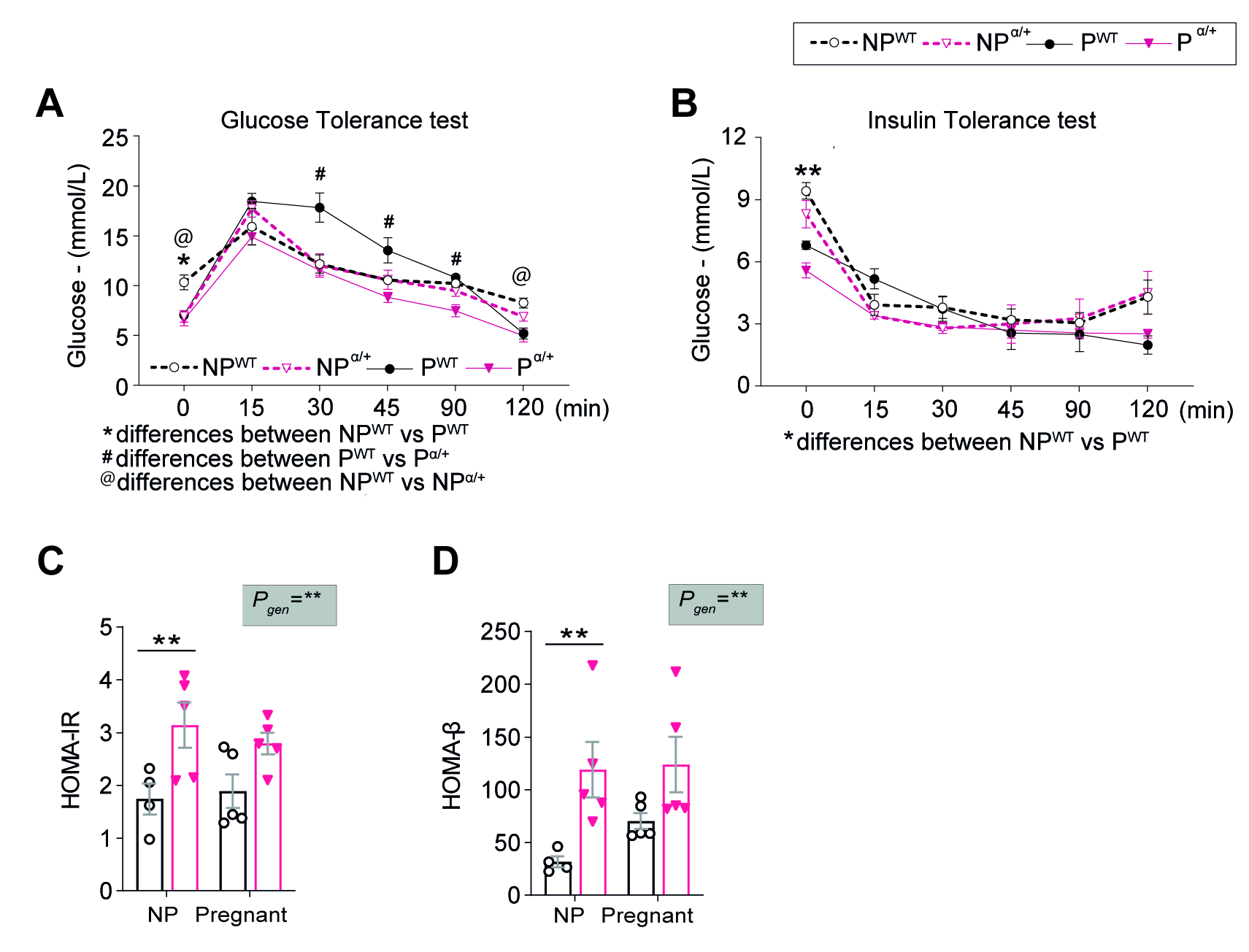

Supplement: Supplementary file 4 [file Image2.TIF]
